# Understanding brain donation decisions: Predictors of indecision and change over time from the Cleveland Alzheimer's Disease Research Center

**DOI:** 10.1002/alz.70698

**Published:** 2025-09-10

**Authors:** Lyndsi Powell, David Silva, Alexsandra Kovacevich, Jagan Pillai, Audrey Lynn, Sunah Song, Brian S. Appleby

**Affiliations:** ^1^ Department of Neurology University Hospitals Cleveland Medical Center Cleveland Ohio USA; ^2^ Department of Neurology Case Western Reserve University School of Medicine Cleveland Ohio USA; ^3^ Center for Brain Health Cleveland Clinic Foundation Cleveland Ohio USA; ^4^ Department of Population & Quantitative Health Sciences Case Western Reserve University School of Medicine Cleveland Ohio USA; ^5^ Cleveland Institute for Computational Biology Case Western Reserve University School of Medicine Cleveland Ohio USA; ^6^ Departments of Psychiatry & Pathology Case Western Reserve University School of Medicine Cleveland Ohio USA

**Keywords:** Alzheimer's disease, autopsy, barriers, brain donation, demographics

## Abstract

**INTRODUCTION:**

Little is known about factors influencing indecision or changes in brain donation program (BDP) enrollment status among Alzheimer's disease and related dementias research participants. This study examined demographic features associated with these decisions in participants from the Cleveland Alzheimer's Disease Research Center (CADRC).

**METHODS:**

Demographics and BDP status were extracted from the CADRC database and analyzed based on initial and current BDP enrollment status.

**RESULTS:**

At baseline, BDP enrollees were more likely to be older, cognitively impaired, White, partnered, have more years of education, and have a relative or partner serving as a study partner. Participants who changed BDP status over time were more often cognitively unimpaired and had more CADRC visits. Fewer years of education predicted changing from “undecided” to “yes.” Enrollment rates varied significantly between study staff.

**DISCUSSION:**

Several demographic features are associated with uncertainty or change in BDP enrollment. These factors should be further examined to optimize ADRC participants’ experience.

**Highlights:**

- Twenty‐one percent of participants changed their brain donation program (BDP) enrollment status during the study.
- BDP enrollment changers were more often cognitively normal and had more study visits.
- Fewer years of education predicted switching BDP status from undecided to yes.
- BDP enrollment and change rates varied between interactions with site staff members.

## 1 BACKGROUND

Brain donation plays a crucial role in advancing our understanding of Alzheimer's disease and related dementias (ADRD), providing researchers with invaluable tissue samples for comprehensive studies. The availability of donated brain tissue allows for direct examination of disease pathology and facilitates the development of new diagnostic and treatment strategies.^1^, ^2^

Prior research has shown that multiple factors influence participants’ decisions to enroll in a brain donation program (BDP) in dementia‐related studies, including age, cognitive status, years of education, race/ethnicity, religious and cultural beliefs, family involvement, and communication between clinicians and caregivers.^1^, ^3^, ^4^, ^5^, ^6^, ^7^ Prior studies have identified group‐specific concerns among individuals who have consented to brain donation, such as greater worry among older Black and Latino adults about family members not following through on their donation wishes, and uncertainty among lower‐income, White adults regarding the logistics of the autopsy process. However, little is known about how these uncertainties may evolve over time.^8^ Furthermore, Black and Latino participants donate at lower rates than non‐Latino White participants, underscoring the need for inclusive family involvement and culturally sensitive communication to address barriers across diverse populations.^2^, ^4^, ^9^ Cultural and religious beliefs have also been shown to influence participants’ decisions regarding brain donation. For example, individuals from Black and Latino communities may cite traditions or values such as maintaining bodily integrity after death or viewing the body as sacred, which can present barriers to donation.^5^, ^10^, ^11^

Additionally, fewer years of education and cognitive impairment have been associated with increased withdrawal rates from brain autopsy programs.^6^, ^12^ Family involvement is another crucial factor in both initial consent and long‐term commitment to brain donation, as family disagreement often results in BDP withdrawal.^6^, ^8^ Participants who have lost a spouse or who live alone are significantly less likely to proceed with donation, as they may lack a close family member to ensure their wishes are fulfilled.^6^ Furthermore, interactions with specific study staff members can play a notable role in participants’ decisions. Research indicates that certain staff members are more successful in obtaining consent, emphasizing the impact of interpersonal communication and trust in the decision‐making process.^1^ Prior research also indicates that there are misconceptions regarding brain autopsy whereby individuals believe that their registration as an organ donor includes brain autopsy, thus highlighting the need for more education on the topic as a whole.^13^

Previous studies suggest that decision making around brain donation is not static; it can be influenced by ongoing discussions with family members, evolving attitudes toward research, and repeated interactions with study personnel.^1^, ^3^ However, there is limited research examining how demographic characteristics, study participation experiences, and personal factors influence individuals who remain uncertain about brain donation or later modify their commitment.^4^, ^11^ Investigating these aspects could help research centers develop more tailored approaches to participant engagement, ensuring that individuals have the information and support they need to make informed decisions.^5^, ^14^

This study aims to address these gaps by examining demographic and study‐related factors associated with indecision about brain donation and changes in brain donation status over time among participants in the Cleveland Alzheimer's Disease Research Center (CADRC). We seek to identify demographic characteristics associated with being undecided about brain donation at initial enrollment and investigate factors that predict changes in brain donation status, specifically those who were initially undecided. Additionally, we will assess the role of study interactions, including the influence of specific study personnel, in shaping participants’ decisions about brain donation. By analyzing these factors, we hope to contribute to a more nuanced understanding of brain donation decision making and provide actionable recommendations for Alzheimer's Disease Research Centers (ADRCs) seeking to improve participant retention and engagement in BDPs.

## 2 METHODS

All data for this study were queried from the CADRC database. The CADRC has collected longitudinal, observational data from a diverse cohort of research participants as part of a national initiative coordinated by the National Institute on Aging (NIA) through the National Alzheimer's Coordinating Center (NACC) since 2019. For the purpose of these analyses, we included all participants enrolled at the University Hospitals (UH) and Cleveland Clinic (CCF) sites of the CADRC from study launch (May 24, 2019) through September 30, 2024. Each participant undergoes an annual consensus diagnostic review to determine or update cohort classification. Consensus review is completed by a panel of clinical experts who discuss relevant exam and biomarker data (detailed below) to provide the most appropriate clinical diagnosis. Participants must be at least 18 years old at enrollment and must have a designated “study partner” who is also > 18 years old. Study partners serve as informants regarding the participant's cognitive function, memory, and activities of daily living. The CADRC actively seeks a local representative population in participant enrollment across sex, race/ethnicity, age, and diagnostic category.

Data were obtained through routine observational study procedures, including neuropsychological testing, structured questionnaires (e.g., medical and family history, medication review, demographics), physical examinations, biofluid collection for both cerebrospinal fluid and plasma biomarkers, neuroimaging, and neuropathological assessments. Neuropathological data were derived from participants who enrolled in the BDP, which is offered to all CADRC participants, at their baseline visit and at each subsequent annual visit. BDPs were affiliated with the CADRC and studies dual enrolling with the CADRC, such as the Dementia with Lewy Body Consortium (DLBC); however, some participants were enrolled in outside programs. For this study, a participant was considered enrolled in a BDP regardless of the program's affiliation. All patients in this study were seen through the CADRC. Autopsy results from the BDP are shared with the deceased participant's next of kin by the study principal investigator ≈ 3 months after the procedure.

Statistical analyses were conducted using SPSS software (version 29) and *χ* ^2^ analyses were used to examine differences in categorical variables, and analyses of variance were applied to continuous variables. Statistical significance was defined as a *P* value < 0.05. Measures of effect are included in the Results section for statistically significant findings. These analyses aimed to evaluate demographic and clinical differences in brain donation status at baseline and to identify characteristics of participants who changed their brain donation status over time (referred to as “changers”).

For participants enrolled at the UH site, data regarding the specific staff member who conducted the brain donation consent were collected manually through a review of consent forms from both baseline and most recent study visits. These data were analyzed to explore whether individual staff members who obtained consent influenced BDP enrollment rates. However, staff characteristics such as years of experience or knowledge of brain donation were not assessed, as complete data were not available for all consenters. Research coordinators introduce the BDP option during the (re)consent process at each annual visit. The topic may be revisited later during the annual visit by the clinician or between visits by research coordinators, though this varies. While the consenting approach is not standardized and varies by staff member, all participants receive a printed FAQ packet addressing common concerns, including clarification that costs related to autopsy and transportation are covered by the study.

Brain donation status at baseline and at the most recent study visit was categorized into one of three groups: “yes” (enrolled), “no” (declined), and “undecided.” Participants were classified as “changers” if their donation status changed between the baseline and most recent study visit. Six switching patterns were defined: yes to no; yes to undecided; no to yes; no to undecided; undecided to yes; and undecided to no.

**RESEARCH IN CONTEXT**

1. **Systematic review**: We reviewed prior studies on brain donation decisions in Alzheimer's disease and related dementias research. Previous studies highlight the impact of race, age, cognitive status, education, and religious/cultural beliefs on decisions to participate in brain donation programs. However, relatively few studies have examined indecision or tracked changes in donation status over time.
2. **Interpretation**: Our findings demonstrate that brain donation decisions are not static. Family involvement, repeated engagement with research teams, and individual demographics may impact the decision to enroll in a brain donation program or change enrollment status over time. Interactions with staff may also impact likelihood to enroll or change status over time.
3. **Future directions**: More research is needed to understand qualitatively why participants may delay or decline consent. There is a need to better understand how staff training and communication strategies affect donation decisions over time.

Key variables included in the analyses were: age, sex, race/ethnicity, years of education, marital status, study partner relationship (defined as a relative, including spouse/partner, or non‐relative), diagnostic cohort at baseline and most recent visit, most recent biomarker results, and total number of completed study visits.

## 3 RESULTS

### 3.1 Demographics

A total of 283 participants were identified in the CADRC database. Six participants were excluded due to incomplete data regarding BDP enrollment status. The final analytic sample included 277 participants. Among the participants included in this study, 131 (47%) were female and 146 (53%) were male. The majority identified as White (*n* = 226, 82%), followed by Black (*n* = 46, 17%), Asian (*n* = 2, 1%), and other (*n* = 3, 1%). Nine participants (3%) identified as Hispanic. The mean age at the baseline and most recent visits were 68 years (standard deviation [SD] = 10.5) and 69 years (SD = 10.7), respectively. Most participants were married or partnered (*n* = 196, 71%), and the mean years of education was 16 years (SD = 2.7). On average, participants completed 2.5 visits (SD = 1.4).

After consensus diagnostic review at the most recent visit, 107 participants (39%) were cognitively normal. Among the 54 participants with mild cognitive impairment (MCI), 35 (13%) had MCI due to Alzheimer's disease (MCI‐AD), 2 (1%) had atypical AD (MCI‐AtAD), 5 (2%) had dementia with Lewy bodies (MCI‐DLB), 1 (0.5%) had frontotemporal lobar degeneration (MCI‐FTLD), and 11 (4%) had other MCI types. Of the 93 participants with dementia, 44 (16%) had AD dementia, 14 (5%) had atypical AD, 10 (4%) had Down syndrome‐related AD, 24 (9%) had DLB dementia, and 1 (0.5%) had FTLD dementia. Additionally, 7 (3%) participants were classified as “other” and 16 individuals had not yet undergone consensus diagnostic review at the time of this data query or had withdrawn prior to completing the data collection used for cohort classification. Among 21 deceased participants, 16 (76%) completed a brain autopsy. See Table 1 for full demographic and diagnostic details.

**TABLE 1:** Participant demographics.

Participant demographics	*N* (%)
Female	131 (47%)
Race	
White	226 (82%)
Black	46 (17%)
Asian	2 (1%)
Other	3 (1%)
Hispanic	9 (3%)
Married/partnered	196 (71%)
Current diagnosis (*n*, %)	
Cognitively normal	107 (39%)
MCI‐AD	35 (13%)
MCI‐AtAD	2 (1%)
MCI‐DLB	5 (2%)
MCI‐FTLD	1 (0.5%)
MCI‐other	11 (4%)
AD dementia	44 (16%)
AtAD dementia	14 (5%)
DS‐AtAD dementia	10 (4%)
DLB dementia	24 (9%)
FTLD dementia	1 (0.5%)
Other	7 (3%)
Years of education (mean, SD)	16 (2.7)
Number of visits (mean, SD)	2.5 (1.4)
Age at most recent visit (mean, SD)	69 (10.7)

Abbreviations: AD, Alzheimer's disease; AtAD, atypical Alzheimer's disease; DLB, dementia with Lewy bodies; DS, Down syndrome; FTLD, frontotemporal lobar degeneration; MCI, mild cognitive impairment; SD, standard deviation.

### 3.2 Baseline enrollment data

At baseline, 113 participants (41%) elected to enroll in the BDP, 25 (9%) declined, and 139 (50%) were undecided. Participants who enrolled at baseline were more likely to be partnered (odds ratio [OR]: 1.23, 95% confidence interval [CI]: 1.06–1.41), cognitively impaired (OR: 1.38, 95% CI: 1.10–1.71), White (OR: 1.17, 95% CI: 1.05–1.30), and have a relative as a study partner (OR: 1.11, 95% CI: 1.03–1.21). They were also older (mean age 70 ± 8 years, mean difference: 3.82 years, 95% CI: 1.46–6.17) and had more years of education (mean 17 ± 3 years, mean difference: 0.79, 95% CI: 0.14–1.44) compared to those who did not enroll (undecided or no). See Table 2 for full details regarding enrollment status at baseline visit.

**TABLE 2:** Demographic data and brain donation enrollment status at the initial study visit.

	Initial (baseline) visit donation status			
	Agreed (112, 41%)	Declined (24, 9%)	Undecided (138, 50%)	*P* value
Female (*n*, %)	44 (34)	13 (10)	74 (57)	0.069
Race (*n*, %)				
White	101 (45)	18 (8)	107 (47)	**0.018**
Black	11 (24)	7 (15)	28 (61)	
Asian	0 (0)	0 (0)	2 (100)	
Other	1 (33)	0 (0)	2 (67)	
Hispanic (*n*, %)	2 (22)	1 (11)	6 (67)	N/A
Married/partnered (*n*, %)	90 (46)	16 (8)	90 (46)	**0.031**
Cognitively normal (*n*, %)	42 (32)	11 (9)	77 (59)	**0.013**
Study partner is a relative or partner (*n*, %)	107 (44)	20 (8)	116 (48)	**0.04**
Mean age at enrollment (mean, SD)	70 (8)	66 (12)	66 (12)	**0.011**
Mean years of education (mean, SD)	17 (2.6)	15 (2.8)	16 (2.6)	**0.003**
Mean CSF Aβ42/40 ratio (SD)	0.18 (0.09)	0.16 (.04)	0.190 (0.07)	0.352
Mean CSF p‐tau (SD)	119 (82)	126 (113)	114 (93)	0.886
Mean Centiloid score (SD)	36 (43)	25 (32)	28 (39)	0.364

Abbreviations: Aβ, amyloid beta; CSF, cerebrospinal fluid; p‐tau, phosphorylated tau; SD, standard deviation.

Statistically significant findings (*p* < 0.05) are bolded.

### 3.3 Participants who changed BDP enrollment status

At the most recent visit, 144 participants (52%) were enrolled in the BDP, 40 (14%) had declined, and 93 (34%) were undecided. In total, 56 participants (20%) changed their BDP status, of whom 49 (88%) were undecided at baseline. “Changers” were more likely to be cognitively normal (OR: 1.67, 95% CI: 1.26–2.22) and had completed more visits on average (mean = 3 ± 1, mean difference: 0.58, 95% CI: 0.23–0.94).

Among those who were undecided at baseline and later switched, participants who eventually enrolled had significantly fewer years of education (mean 16 ± 2 years, mean difference: −1.65, 95% CI: −3.05 to −0.25) compared to those who ultimately declined (mean 18 ± 2 years). Only three participants (5% of changers) changed from enrolled at baseline to a different status, but the group was too small for meaningful statistical analysis (see Table 3).

**TABLE 3:** Demographic features are displayed for patients who changed their donation status throughout the course of the study.

Changed donation status						
	Changed (56, 20%)	Unchanged (224, 80%)	*P* value	Undecided to yes (32, 65%)	Undecided to no (17, 35%)	*P* value
Female (*n*, %)	27 (21)	104 (79)	0.877	17 (71)	7 (29)	0.426
Race (*n*, %)						
White	50 (22)	78 (78)		29 (66)	15 (34)	
Black	3 (18)	43 (64)		2 (100)	0 (0)	
Asian	2 (100)	0 (0)		1 (50)	1 (50)	
Other	1 (33)	2 (67)		0 (0)	1 (100)	
Hispanic (*n*, %)	3 (33)	6 (67)	0.718	0 (0)	2 (100)	
Mean age at most recent visit (SD)	72 (11)	69 (11)	0.085	70 (13)	73 (8)	0.511
Married/partnered (*n*, %)	37 (19)	159 (81)	0.361	21 (62)	13 (38)	0.433
Cognitively normal (*n*, %)	32 (30)	75 (70)	**0.001**	20 (71)	8 (29)	0.466
Study partner is a relative or partner (*n*, %)	46 (19)	197 (81)	0.154	25 (64)	14 (36)	0.697
Years of education (mean, SD)	16 (2)	16 (3)	0.136	16 (2)	18 (2)	**0.022**
Number of visits (mean, SD)	3 (1.3)	2 (1.3)	**0.001**	3 (1)	3 (1)	0.279
CSF Aβ42/40 ratio (mean, SD)	0.183 (0.06)	0.180 (0.08)	0.522	0.195 (0.06)	0.187 (0.06)	0.685
CSF p‐tau (mean, SD)	114 (92)	119 (88)	0.769	113 (97)	98 (69)	0.622
Centiloid score (mean, SD)	23 (39)	35 (40)	0.113	23 (42)	26 (39)	0.798

*Note*: We compared participants who changed their status to participants who did not change their status. We then more specifically compared participants who were originally “undecided” and changed their status to “yes” to those who were originally “undecided” and changed status to “no.”

Abbreviations: Aβ, amyloid beta; CSF, cerebrospinal fluid; p‐tau, phosphorylated tau; SD, standard deviation.

Statistically significant findings (*p* < 0.05) are bolded.

### 3.4 Consenting staff

Enrollment success appeared to differ significantly by the research staff member who obtained consent for BDP. At the UH site, one staff member (referred to as the “super‐consenter”) was found to have a significantly lower proportion of undecided participants at baseline (22%) compared to the remaining staff (79%, OR: 0.47, 95% CI: 0.25–0.89). At the baseline visit, participants enrolled in the BDP more frequently with this staff member than all the other staff members combined (OR: 2.27, 95% CI: 1.19–4.33).

## 4 DISCUSSION

This study aimed to better understand patterns of brain donation enrollment and identify factors associated with indecision and changes in donation status over time. At baseline, approximately half of participants (50%) were undecided about brain donation, highlighting the complexity of consent decisions in ADRD research and brain donation. Our findings support existing literature showing that brain donation decisions are dynamic and can evolve in response to ongoing discussions, cognitive changes, life events, and study‐related experiences.^4^, ^15^ Notably, 20% of participants in this study changed their brain donation status after their baseline visit, emphasizing the need for sustained engagement and reevaluation of consent throughout participation. Additionally, initial and current BDP enrollment status (41% and 52%, respectively) were not an accurate predictor of overall autopsy rate (76%).

Participants who enrolled in the BDP at baseline were more likely to be older, cognitively impaired, White, partnered, have more years of education, and have a relative or partner serving as a study partner. These findings are consistent with prior research that associates these demographic characteristics with increased willingness to donate.^3^, ^4^ Family involvement appears particularly impactful, aligning with studies that highlight the crucial role of caregivers and relatives in donation decisions.^6^, ^16^ Brain autopsy results offer family members neuropathological data that may directly impact their future health and outcomes. This alone could be a motivating factor not only for the participant's decision to enroll in a BDP, but also for the family's support regarding enrollment and subsequent follow‐through with autopsy.^1^ These baseline findings also align with prior findings that have shown those who live alone or have lost a spouse are less likely to donate, possibly because they lack a family member who can oversee the process.^6^ Furthermore, participants with cognitive impairment were more likely to consent at baseline, which we hypothesize may be due to increased recognition of the value of their contribution to dementia research or encouragement and support from their caregivers.

Interestingly, participants who were undecided at baseline and later enrolled in the BDP had fewer years of education than those who ultimately declined. This contrasts with prior findings suggesting that fewer years of education is associated with withdrawal or refusal.^12^ One potential explanation is that repeated contact with research staff and growing familiarity with the study may mitigate initial hesitancy among participants with fewer years of education, which would support the benefit of longitudinal, relationship‐centered engagement strategies. Further research should explore the specific factors that contribute to eventual enrollment among initially undecided participants with fewer years of education.

Race and ethnicity were also significantly associated with donation decisions. White participants were more likely to enroll in the BDP than participants of other racial backgrounds, reflecting disparities documented in previous studies.^2^, ^4^, ^6^ Barriers to brain donation among Black and Latino populations are well documented and include concerns about respect for bodily integrity, mistrust in medical systems, and uncertainty about family follow‐through.^3^ Some cultural traditions also emphasize maintaining body wholeness after death, which can discourage donation.^3^, ^5^ Addressing these concerns requires culturally sensitive approaches that incorporate family dialogue, respect for cultural beliefs, and community outreach led by trusted messengers.

Another notable finding was the significant influence of one UH staff member who conducted baseline consents. Participants consented by a single “super‐consenter” were significantly more likely to have made a decision (yes or no) rather than remain undecided and were more likely to enroll in the BDP. Though this super‐consenter staff member had a PhD in a non‐clinical biologic science, they had comparable clinical research experience to other staff members obtaining consent; however, complete data regarding years of experience was unavailable to fully assess. Furthermore, BDP enrollment decisions varied significantly across consenting staff. Prior research has highlighted the role of interpersonal trust and communication style in shaping participant engagement and willingness to consent.^1^, ^5^ While these specific factors could not be evaluated in the current analysis, their potential influence warrants further investigation. Future studies should examine staff characteristics and consenting strategies to identify which interpersonal or procedural elements most effectively support informed, affirmative BDP decisions. Participants who changed their brain donation status over time, regardless of the direction, were more likely to be cognitively normal and to have completed more study visits. This finding supports the notion that decision making around brain donation evolves with increased familiarity with the research process, potentially reflecting growing trust in the research team or shifting personal values.^2^, ^15^ These results echo prior work suggesting that continued participation and communication help build confidence and comfort with donation decisions over time.^10^

Interestingly, we found that sex, biomarker data, and etiologic diagnosis did not predict enrollment status or change in decision making. This suggests that other factors, especially those related to interpersonal dynamics and lived experience, may be more critical in influencing brain donation decisions than biological or clinical characteristics.

### 4.1 Implications

Findings from this study offer several actionable insights for ADRD‐centered programs seeking to improve brain donation enrollment:

1. **Longitudinal engagement is essential**: Participants’ decisions often evolve over time, particularly among those who are initially undecided. Regular check‐ins, educational reinforcement, and opportunities to revisit the donation decision can promote informed commitment.
2. **Family involvement should be prioritized**: Spousal or familial participation during the consent process improves enrollment and follow‐through. Facilitating family discussions and addressing caregiver concerns directly can improve decision confidence and clarity.
3. **Culturally tailored communication is critical**: Underrepresented groups express unique concerns about brain donation. BDPs should integrate community‐informed approaches, involve culturally concordant staff, and respect religious and cultural values to foster trust and inclusivity.
4. **Staff training can significantly impact outcomes**: The success of specific staff in gaining participant commitment supports the value of staff engagement when making such decisions. Further studies and initiatives should focus on administering training programs that emphasize empathy, cultural competence, and clear communication.
5. **Ongoing uncertainty should be addressed explicitly**: With a large portion of participants being undecided at baseline and remaining so at later visits, tailored interventions, including informational materials, testimonials, and structured discussions, should be offered early and revisited throughout study participation.
6. **BDP enrollment may not be an accurate predictor of brain donation**: BDP enrollment is often viewed as a predictive metric for neuropathology core autopsies. In our cohort, enrollment in the BDP was not a good predictor and underestimated the number of eventual brain donations. This should be examined in other research programs, and other predictive metrics should be considered if this finding is validated.

### 4.2 Limitations

Several limitations should be considered when interpreting these findings. First, this study did not have access to consistent qualitative data on participants’ specific reasons for declining or delaying BDP enrollment. Notably, we lacked direct information on religious or cultural beliefs influencing decision making. Given that such beliefs have been shown to play a substantial role in donation decisions,^3^, ^5^ future studies would benefit from collecting detailed participant‐reported barriers and motivations. Additionally, there were limited data regarding those who changed their BDP enrollment status multiple times throughout their time in the study. Addressing this group of participants with qualitative analysis would offer insight into the nuances regarding participant decision making as it relates to brain donation.

Second, the sample included those who were predominantly White and had more years of education, limiting generalizability to more diverse populations. Although this reflects the demographics of many research cohorts, future research should aim to include more participants from historically underrepresented groups to better understand culturally specific barriers and facilitators.

Third, while we identified staff‐specific differences in enrollment success, we were unable to fully assess staff characteristics (e.g., years of experience, training background, communication style, or staff knowledge regarding brain donation) that may explain these disparities. This limitation was partially due to the unavailability of former staff members involved in the consenting process, which restricted access to these data. More detailed analysis of staff–participant interactions could further illuminate mechanisms that build trust and promote consent.

## 5 CONCLUSION

This study adds to the growing body of research on brain donation decision making by highlighting the importance of demographic, relational, and study‐process factors in both initial consent and changes in consent status to brain donation over time. Our findings underscore that indecision is common, but not fixed, and that ongoing engagement, family involvement, and staff interactions can influence participants’ willingness to commit to brain donation. Future efforts should focus on improving consent processes and addressing barriers for underserved populations to ensure equitable participation in brain donation and the broader research enterprise it supports.

## AUTHOR CONTRIBUTIONS

Lyndsi Powell: collection of data, analysis of data, writing of manuscript; David Silva: analysis of data, writing of manuscript; Alexsandra Kovacevich: analysis of data, editing of manuscript; Jagan Pillai: collection of data, editing of manuscript; Audrey Lynn: analysis of data, editing of manuscript; Sunah Song: analysis of data, editing manuscript; Brian S. Appleby: study design, collection of data, analysis of data, editing of manuscript.

## CONFLICT OF INTEREST STATEMENT

The authors declare that they have no conflicts of interest related to the research described in this manuscript. Author disclosures are available in the supporting information.

## CONSENT STATEMENT

The CADRC study is approved by the Cleveland Clinic Foundation and the University Hospitals Cleveland Medical Center's Institutional Review Boards. The study is performed in accordance with the ethical standards as laid down in the 1964 Declaration of Helsinki and its later amendments.

## Supporting information



Supporting information
